# Genomic Variation Shaped by Environmental and Geographical Factors in Prairie Cordgrass Natural Populations Collected across Its Native Range in the USA

**DOI:** 10.3390/genes12081240

**Published:** 2021-08-13

**Authors:** Jia Guo, Patrick J. Brown, Albert L. Rayburn, Carolyn J. Butts-Wilmsmeyer, Arvid Boe, DoKyoung Lee

**Affiliations:** 1 Department of Forest Ecosystems and Society, Oregon State University, Corvallis, OR 97331, USA; jia.guo@oregonstate.edu; 2 Department of Plant Sciences, University of California at Davis, Davis, CA 95616, USA; pjbrown@ucdavis.edu; 3 Department of Crop Sciences, University of Illinois, Urbana, IL 61801, USA; arayburn@illinois.edu (A.L.R.); cjbutts2@illinois.edu (C.J.B.-W.); 4 Department of Agronomy, Horticulture, and Plant Science, South Dakota State University, Brookings, SD 57007, USA; Arvid.Boe@SDSTATE.EDU

**Keywords:** prairie cordgrass, populations, diversity, variation, SNP, genomics

## Abstract

Prairie cordgrass (*Spartina pectinata* Link) is a native perennial warm-season (C4) grass common in North American prairies. With its high biomass yield and abiotic stress tolerance, there is a high potential of developing prairie cordgrass for conservation practices and as a dedicated bioenergy crop for sustainable cellulosic biofuel production. However, as with many other undomesticated grass species, little information is known about the genetic diversity or population structure of prairie cordgrass natural populations as compared to their ecotypic and geographic adaptation in North America. In this study, we sampled and characterized a total of 96 prairie cordgrass natural populations with 9315 high quality SNPs from a genotyping-by-sequencing (GBS) approach. The natural populations were collected from putative remnant prairie sites throughout the Midwest and Eastern USA, which are the major habitats for prairie cordgrass. Partitioning of genetic variance using SNP marker data revealed significant variance among and within populations. Two potential gene pools were identified as being associated with ploidy levels, geographical separation, and climatic separation. Geographical factors such as longitude and altitude, and environmental factors such as annual temperature, annual precipitation, temperature of the warmest month, precipitation of the wettest month, precipitation of Spring, and precipitation of the wettest month are important in affecting the intraspecific distribution of prairie cordgrass. The divergence of prairie cordgrass natural populations also provides opportunities to increase breeding value of prairie cordgrass as a bioenergy and conservation crop.

## 1. Introduction

Prairie grass species native to North America, such as switchgrass (*Panicum virgatum* L.), big bluestem (*Andropogon gerardii* Vitman), indiangrass (*Sorghastrum nutans* L.), and prairie cordgrass have shown potential use for conservation practices and potential bioenergy production [1,2,3,4]. To develop prairie grass species as a new crop for either conservation practices or bioenergy feedstock on marginal conditions, it is important to characterize and maintain the genetic resources of local or regional populations that show agronomic advantages, such as high biomass yield and strong biotic and abiotic stress tolerances. However, the presence of North American tallgrass prairie has been diminished by agriculture and urban development since European settlement. Although scattered throughout the historical range, thousands of remnant prairie sites still exit in North America [5]. Locally adapted natural populations from those remnant prairie sites are valuable genotypes that harbor adaptive traits to various environments [6,7].

Prairie cordgrass is a native, perennial, warm-season (C4) grass that once dominated North American tallgrass prairies. The habitats of prairie cordgrass cover wet to moist prairies and low areas alongside rivers and tributaries [8,9]. Mobberley [10] found prairie cordgrass also thrive in open, dry prairie and along railroads in the Midwestern United States. Common nursery evaluation of prairie cordgrass in Europe [11], eastern South Dakota [12,13], and central Illinois [1], has shown high biomass yield potential, comparable to that of switchgrass and other warm-season grasses. According to Boe and Lee [12], seven natural populations of prairie cordgrass from South Dakota produced more biomass than switchgrass while showed significant differences for biomass production among populations. Another study evaluating populations collected from an area spanning Midwestern and Eastern USA also showed extensive phenotypic variation among prairie cordgrass populations [1]. Compared to other perennial grasses, such as switchgrass and big bluestem, prairie cordgrass has a limited breeding history, with only five cultivars released as source-identified genetic material [14,15]. The information of genetic background of other prairie cordgrass natural populations is also limited.

Cytotaxonomic studies of prairie cordgrass revealed different ploidy levels existing among populations, including tetraploid (2n = 4x = 40) populations distributed from Southern Canada and the Eastern USA, and octoploid (2n = 8x = 80) populations distributed across Midwestern USA [16,17,18]. A mixed-ploidy population consisting of tetraploid and hexaploid (2n = 6x = 60) individuals was found in central Illinois, USA [19]. Within this mixed-ploidy population, substantial phenotypic variability was observed between two ploidy levels, such as flowering time, stomatal size, and plant morphological characteristics [19]. Kim et al. [20] reported the presence of all three ploidy levels among 11 surveyed natural populations and found that a positive association between genome size and the stomata size between octoploids and tetraploids. A cytogenetic survey of 60 prairie cordgrass natural populations found the tetraploid populations extended from the East North Central to the New England regions of the U.S., and the octoploid cytotypes distributed in the West North Central regions [21]. A study of prairie cordgrass chloroplast DNA (cpDNA) also showed a strong relationship between cpDNA haplotypes and geographic distribution [22]. Three cpDNA haplotype groups including “PCG1” haplotypes occurred in the New England/Middle Atlantic regions in the east and central U.S., a “PCG2” haplotypes found in southern SD and northern IA, IL, and MO in the central U.S., and a “PCG3” haplotypes identified in a distinct region that includes portions of ND, SD, and MN. The major cpDNA haplotype group (“PCG1”) includes members of all three cytotypes. The wide dispersal of cytotypes within cpDNA haplotypes could be resulting from a combination of migration and polyploidization, which is not uncommon in *Spartina* species [23,24].

To fully investigate the genetic variation and phylogeography in this outcrossing species, nuclear molecular markers with single nucleotide polymorphisms (SNPs) should be jointly used with organelle molecular markers [25,26]. With the advent of high-throughput sequencing technology, it is now feasible to survey the whole genome and provide trait-associated molecular markers for phylogenetic studies. DNA libraries constructed using Genotyping-by-sequencing (GBS) on restriction site takes advantages of high-throughput sequencing technology to generate thousands of SNPs across many individuals [27,28]. This simultaneous polymorphism discovery and genotyping approach avoid ascertainment bias while lowering the overall cost by combining many genotypes in a single run [28,29]. A greater number of molecular markers improves clustering of the wild taxa as sources of useful genes in breeding programs and identifies conservation territories of a particular species of interest [30,31,32].

Undomesticated species have often gone through extensive inter-specific gene flow, lineage splitting, and genetic drift, resulting in incongruences of genealogical information carried by each gene [33,34,35]. Environmental and geographical factors are significant contributors in shaping population structure through the above-mentioned divergence events. A better understanding of environmental and geographical adaptation within a species could benefit research communities such as plant breeding, conservation ecology, and evolutionary ecology. Therefore, in this study, we collected 96 prairie cordgrass populations across the east and central midwest US range and genotyped them using a GBS approach. The objectives of this study are to: (1) identify intraspecific genetic diversity among prairie cordgrass natural populations collected in U.S.; (2) reveal the intraspecific bio-geographical distribution of those natural populations.; and (3) evaluate the influences of environmental and geographical variables on the distribution of those populations.

## 2. Materials and Methods

### 2.1. Plant Materials

From 2009 to 2011, seeds of 96 prairie cordgrass natural populations were collected from New England (Maine, Massachusetts, and Connecticut), the Middle Atlantic (New Jersey), the East North Central (Wisconsin, Illinois, and Indiana), the West North Central (Minnesota, Iowa, Missouri, North Dakota, South Dakota, Nebraska, and Kansas), and the West South Central (Oklahoma and Louisiana) regions (United States Census Bureau and Statistical abstract of the United States 2010 edn Washington, DC., https://www.census.gov/geo/reference/gtc/gtc_census_divreg.html, accessed on 28 January 2018) (Table A1). For the best representation of a local population at each location, seeds were collected from all visually identifiable clones within a 1-km radius of the sampling area. When a large cohort of plants were identified, seeds were collected from a random sampling of inflorescences covering the area. Northern Appalachian mountain areas, Ohio, West Virginia, and West New York were searched for prairie cordgrass natural populations in the remnant prairie area. However, there were no prairie cordgrass remnant populations found or reported by the local USDA plant materials collection centers. The county-based USDA-NRCS distribution map also showed a sparse adaptation in those regions for prairie cordgrass (USDA-NRCS, https://plants.usda.gov/core/profile?symbol=sppe, accessed on 29 November 2019). In addition, more than 100 rhizomes of each of two cultivars (‘Kingston’ germplasm (KST), ‘Southampton’ germplasm (STP)) were obtained from the USDA-NRCS Big Flats Plant Material Center, NY. Seeds of ‘Red River’ prairie cordgrass, a cultivar developed by interpopulation open pollination among vegetative propagules obtained from east central Minnesota (Grant County), northeastern South Dakota (Day County), and east central North Dakota (Cass and Grand Forks Counties) [13], were also included in this study (Table 1). Seedlings of four genotypes developed from each population were transplanted on 0.9-m centers in a common field nursery at the University of Illinois Energy Farm in Urbana, IL (40${}^{\circ }$6′ N, 88${}^{\circ }$13′ W). The dominant soil was Drummer silty clay loam (fine-silty, mixed, super-active, mesic typic Endoaquolls). A randomized complete block design with four replications was used to arrange populations. Each plot consisted of 16 plants of the same genotype spaced on 0.9-m centers, and individual plot size was 3.6 m × 3.6 m. Weeds were controlled by applying 0.28 kg ai ha^−1^ quinclorac (3,7-dichloroquinoline-8-carboxylic acid) before the emergence and 0.79 kg ae ha^−1^ 2,4-D ester (2-ethylhexyl ester of 2,4-dichlorophenoxyacetic acid) in the growing season from 2011 to 2013. All plots were also fertilized with 112 kg N ha^−1^ in April of 2011, 2012 and 2013.

### 2.2. Genotyping-by-Sequencing

Leaf tissue samples from each genotype were collected and bulked for DNA extraction in 96-well frozen plant format using a standard CTAB protocol [36]. A minimum of two genotypes from each population were collected. Up to four genotypes were collected when possible. In total, 213 individuals were included in the preparation of sequencing library. DNA was then quantified with PicoGreen (Life Technologies, Grand Island, NY, USA) and prepared for GBS library construction following the proctocol proposed by Poland et al. [28]. Genomic DNA was double-digested using PstI-HF (rare cutting) and HinP1I (common cutting) enzyme. Rare and common restriction overhangs were ligated with two sets of barcoded adapters. Illumina primers (Beckman Coulter, Inc., Indianapolis, IN, USA) were used to amplify pooled restriction ligation reactions. Size of generated fragments were measured using an Agilent Bioanalyzer (Agilent, Santa Clara, CA, USA). The library was submitted to the University of Illinois Keck Biotechnology Center for sequencing on an Illumina Hi-Seq2000 to obtain single-end, 100-bp reads. Raw sequence reads were processed using the GBS-SNP-CROP pipeline [37]. The sequence data were first demultiplexed and trimmed from barcode and cut sites using TRIMMOMATIC [38]. Reads from ten individuals with diverse geographical origins were assembled and clustered to create a pseudo-reference genome using VSEARCH [39]. The diverse set of samples were chosen based on several factors, including the representative populations reported in the previous phylogenetics study using chloroplast sequences, read depth, and ploidy levels. A minimum of 2.5 million reads is required for a sample to be chosen for creating the pseudo-reference genome. An even number of samples were selected from tetra- and octo-ploidy populations Table A1. Processed reads were aligned to the pseudo-reference genome using BWA-mem and SAMtools algorithm to identify all potential SNPs for each sample. Given the high ploidy levels among prairie cordgrass populations and the purpose of this phylogenetic study, SNPs were filtered first based on read depth and then allele frequency. A minimum read depth of 11x is required to call a locus homozygous in the absence of any reads of the alternative for tetraploid or higher levels of ploidy [37]. In addition, the minimum read depth of calling a locus heterozygous is 3x, the required proportion of secondary reads to all non-primary reads is 0.9, the proportion of genotyped individuals to accept a SNP is 0.75, and the acceptable ratio of the depth of the secondary allele to that of the primary allele is 0.1. At last, individuals with read depths lower than 4x were eliminated. Although the read depth filters reduced the number of SNPs retained from the pipeline, it avoided calling SNPs in regions with low coverage. The average read depth for each sample is presented in Table A1. Diploid genotypes were generated in this study to estimate the population structure and heterozygosity for tetra-, hexa-, and octoploids. A study by Bishop et al. [40] indicated all three cytotypes are highly likely allopoly-ploidy by examining the chromosome pairing patterns. However, there was indeed a higher chance for hexa-ploidy to behave differently. Another study by Crawford et al. [41] reported a disomic inheritance based on the distribution of allele frequencies in a bi-parental F1 tetra-ploidy population.

### 2.3. Ploidy Levels

To estimate ploidy level of each population, flow cytometry was performed on the main tiller of four clonally propagated plants collected from one genotype for each population when one or more secondary tillers were initiated. Nuclear DNA content was determined using a procedure modified from Rayburn et al. [42] and Kim et al. [20]. Details on sample preparation were described by Lee et al. [43], and the analysis of relative DNA content was conducted with DB LSR flow cytometry (BD Biosciences, San Jose, CA, USA) in the Flow Cytometry Laboratory (Biotechnology Center, University of Illinois at Urbana-Champaign, Champaign, IL, USA). The relative DNA content was calculated by dividing the relative fluorescence of the sample using the relative fluorescence of the standard. Ploidy level of each population was determined according to Kim et al. [20]. Briefly, a plant sample with 1.6 picogram (pg) DNA content would be designated tetraploid (2n = 4x), a 2.3 pg plant would be considered to be hexaploid individual (2n = 6x), while the 3.1 pg plants would represent octoploid plants (2n = 8x).

### 2.4. Population Structure and Genetic Diversity

For population and genetic diversity analyses, we selected data from one genotype from each population to represent each population. Samples with higher than 15% missing rate were also avoided. A total of 96 samples were selected. Single nucleotide polymorphisms data were first imputed using the LD-kNNi algorithm in Tassel V5 [44,45] and scored in a binary format as homozygous primary allele (0), heterozygous (1), and homozygous secondary allele (2). The LD-KNNi algorithm is based on a k-nearest neighbor genotype imputation method, designed for unordered markers on unphased genotype data from heterozygous species. Population structure was analyzed using *fastSTRUCTURE*, a Bayesian-based algorithm [46], and the discriminant analysis of principal components (DAPC, ‘adegenet’ package, R Development Core Team 2013) [47] to visualize the genome-wide patterns of distribution and potential group membership of each population. The *fastSTRUCTURE* was run from K = 1 to K = 10 using default parameters for 96 samples. Geographical distribution of populations was mapped on the US state map using ggplot (‘ggplot2’) [48] based on the coordinates of collection origins. We also evaluated the likelihood provided by *fastSTRUCTURE* and the Bayesian information criterion (BIC) score provided by *DAPC* to infer the best number of demes supported by the data Figure A2. The principal coordinate analysis (PCOA) was then performed using pcoa (‘ape’ package) [49] to investigate the genetic differentiation among and within demes. An analysis of molecular variance (AMOVA) was conducted on all individuals for genetic variation associated with ploidy levels, populations, demes, and plants using the poppr.amova (‘poppr’ package) [50] in R. We proposed two models. In the first model, we explored the genetic variation at levels of ploidy, populations within each ploidy, and samples within each population. This provides information of genetic variation for a plant breeder to perform selections within and among potential landraces. In the second model, we evaluated the effects of levels of demes, ploidy levels within demes, and populations within ploidy. Since we imposed the category of demes in the second model, results from second model are approximates of levels of variance explained by demes, ploidy levels, populations, and genotypes. Heterozygosity and fixation statistics were calculated for within and among potential genetic groups using genet.dist (‘hierfstat’ package) [51] in R according to Weir & Cockerham [52].

### 2.5. Environmental and Geographical Variables

To assess the association of environmental and geographical variation with the genetic variation of natural prairie cordgrass populations, a 30-year normals for temperature and precipitation were collected from National Oceanic and Atmospheric Administration (NOAA) weather stations located closest to the collection site (https://www.ncei.noaa.gov/products/us-climate-normals, accessed on 16 May 2019). Three geographical variables are longitude (LONG) and latitude (LAT) (expressed in hundredths of degrees) and altitude (ALT) (expressed in meter). The environmental variables were then calculated to generate more biologically meaningful variables using a ’biovar’ function in the R package ‘dismo’ [53]. The values of each variable were converted using log-transformation based on a Box-Cox transformation test to promoting normality (Shapiro–Wilk tests: *p* > 0.05). A total of 17 environmental variable including an EcoregionIII Factor (EF), 8 temperature variables and 8 precipitation variables over 30 years (from 1987 to 2017) were collected. The EF was created according to Omernik [54], who defined a local ecosystem for its quality and integrity, by evaluating its pattern and composition of biotic and abiotic phenomena. The ambient temperature variables (expressed in °C) are mean annual temperature (MAT), standard deviation of annual temperature (SDAT), mean temperature of the warmest month (MTWM), and mean temperature of the coldest month (MTCM). We also collected mean temperature of each of four meteorological seasons: Spring (1 March– 31 May), Summer (1 June–31 August), Autumn (September 1st–November 30th), and Winter (1 December–28 February), expressed as MTSP, MTSU, MTAU, and MTWI, respectively. The precipitation variables (expressed in mm) were collected in the same way as the temperature variables, hence mean annual precipitation (MAP), standard deviation of annual precipitation (SDAP), mean precipitation of the wettest month (MPWM), mean precipitation of the driest month (MPDM), and mean precipitation of Spring (MPSP), Summer (MPSU), Autumn (MPAU), and Winter (MPWI).

### 2.6. Mantel Tests and Canonical Correlation Analyses

To evaluate the influence of environmental adaptation on the formation of subgroups within prairie cordgrass, we conducted mantel tests and canonical correlation analyses using the SNP data, environmental and geographical data. For mantel tests, the environmental distance matrix was created based on 17 environmental variables, using the vegdist function in ‘vegan’ package in R [55,56]. To create a geographical distance matrix among populations, we calculated pair-wise geographical distances based on latitude/longitude degrees on an ellipsoidal model of the Earth, also known as the method of Vincenty’s Formulae [57], using the gdist function under ‘Imap’ package in R [58]. Genetic distance (*F_ST_* values) matrix was calculated using the genet.dist function in ‘hierfstat’ package, in which the Weir & Cockerham approach was used [52]. The mantel tests were carried out using the ‘vegan’ package in R [56]. Significance testing of the correlations was performed with 10,000 permutations. In this study, we conducted Mantel test of correlations of both environmental and geographical distance with genetic distance. In addition, a Partial Mantel test was run between environmental and genetic distance, while controlling for geographical distance. Although Mantel test is popular in landscape genetics studies, it provides low detecting power in studying relationships between distance matrices and lacks ability to estimate proportional contribution of variation from environmental and geographical variables [55,59,60,61]. Therefore, we conducted canonical correlation analyses (CCA) following Mantel tests to evaluate the rank of importance of environmental and geographical variables in contributing to the genetic variation within the species [62,63]. In order to reduce the dimensionality of genomic data while retaining information covering the whole genome, we chose the first 10 PCOA axes from the PCOA of the SNP data (a total of 50.7% variance explained, data not shown) for the CCA. All 20 environmental and geographical variables were used for creating pair-wise canonical variables with the 10 PCOA axes. The CCA first decomposed the variance contributed by each canonical variable, and then calculated the correlations between environmental/geographical variables with the selected canonical variables. The canonical correlation analysis was carried out using cc function in the ‘CCA’ package [64]. The statistical significance of canonical correlation coefficients was examined using *F*-approximations of Wilks’ Lambda using p.asym function in ‘CCP’ package [65].

## 3. Results

### 3.1. SNP Discovery

**Figure A1:**
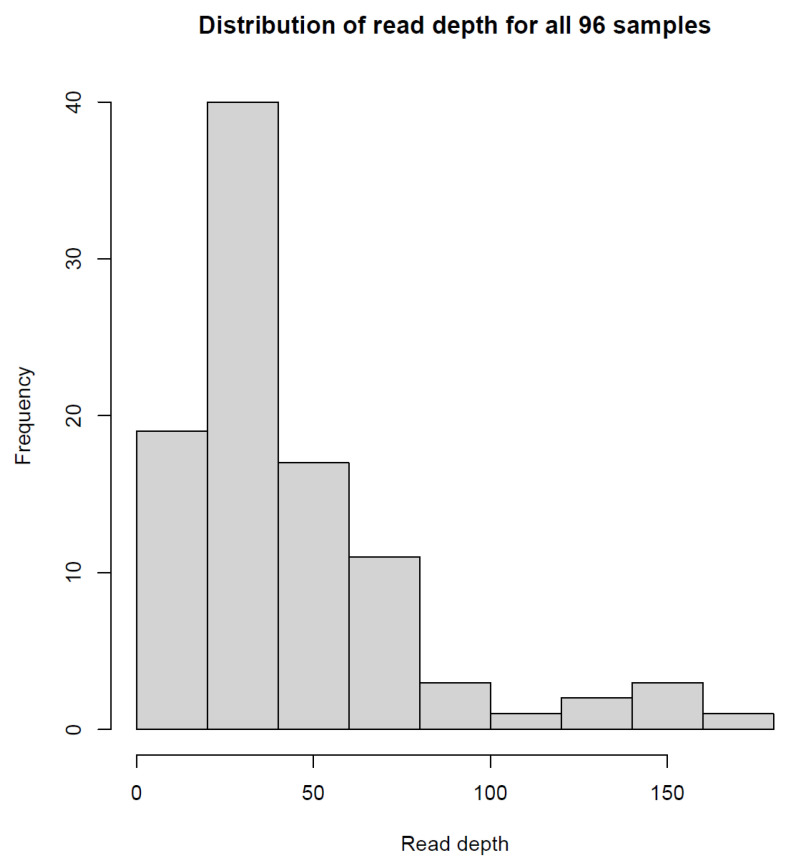
Graph presenting distribution of mean read depth for all 96 samples genotyped using 9315 SNPs with a GBS approach. In the *x*-axis, read depth data including primary and secondary alleles are presented. In the *y*-axis, the frequency of samples with corresponding read depth is presented.

A total of 240 million single-end sequence reads were produced from the Illumina Hi-Seq2000 platform. The minimum and maximum lengths were 32 and 90 base pairs (bp), respectively. A total of 29.7 million reads were used to build the pseudo-reference genome based on available computational power. The final pseudo-reference genome contains 371,332 sequences and 19.1% of them were non-singletons which were filtered in the downstream analyses. The average length of the reference sequences was 82 bp with a standard deviation of 13.5 bp. The initial assembly and SNP calling without filters yielded 211,294 SNPs. A final subset of 9315 SNPs was retained and genotyped in 213 samples after applying restrictions on read depths and allele frequencies. The average read depth was 36.4 × per SNP. The distribution of sample read depth is also provided Figure A1. Individuals on average had 19.8% and 8.9% missing SNPs before and after imputation, respectively.

### 3.2. Ploidy Levels

**Figure 1:**
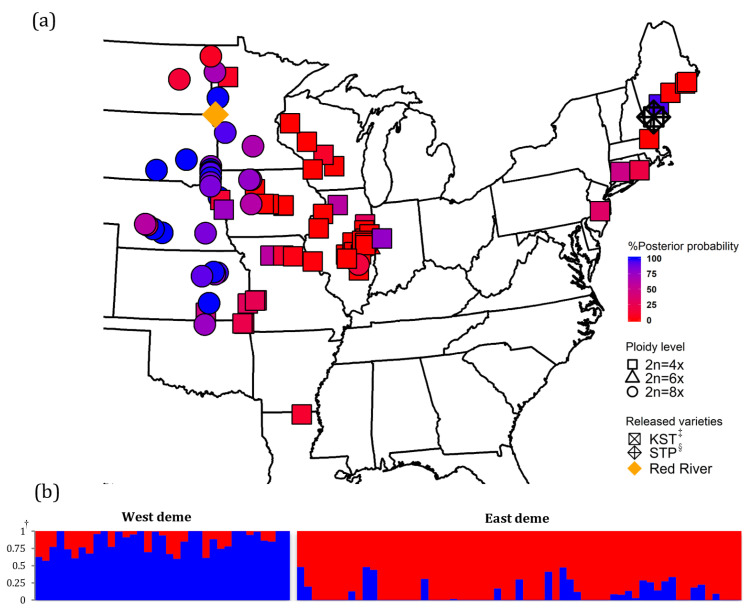
Geographical distribution of prairie cordgrass collections. (**a**) Map of collection in native range. Three shapes correspond to three levels of ploidy as indicated by the legend. Rectangle, circle, and triangle represent tetraploids, octoploids, and hexaploids, respectively. The populations were colored in a gradient scale based on the probability of membership assigned to two groups. (**b**) Bar charts showing posterior probabilities of assignment to two groups based on algorithms of variational Bayesian framework (*fastStructure*) and discriminant analysis of principal components (*DAPC*) using 9315 SNPs data. ^†^: Bayesian-based posterior probability calculated from *fastStructure* and *DAPC*; ^‡^: KST = Kingston germplasm, Big Flats plant material center, NY; ^§^: STP = Southampton germplasm, Big Flats plant material center, NY.

There were three DNA ploidy levels found in this study: tetraploid, hexaploid, and octoploid cytotypes (Table A1). The intraspecific ploidy level variation was congruent to the results from Kim et al. [21]. In this study, 56 populations were classified as tetraploids (2n = 4x), 4 populations were classified as hexaploids (2n = 6x), and 36 populations were classified as octoploids (2n = 8x). Most of the tetraploids were identified in the East North Central and New England U.S. regions (CT, NJ, MA, ME, IN, MO, and LA) (Figure 1). All four hexaploids were identified in Illinois. The majority of octoploids were identified in the West Central region (SD, NE, and ND). Two different ploidy levels were identified in OK, NY, MN, KS, IL, WI, and IA.

### 3.3. Population Structure

**Figure A2:**
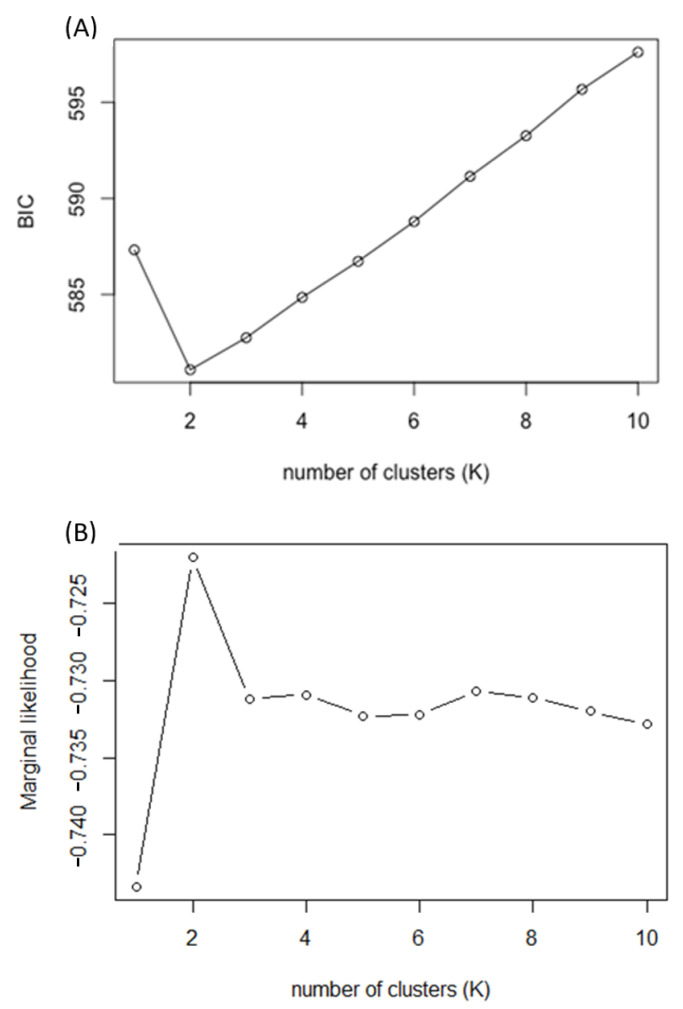
(**A**) Graph showing number of potential genetic clusters (*K*) associated with value of Bayesian Information Criterion (BIC) calculated using *DAPC* algorithm. (**B**) Graph showing number of potential genetic clusters (*K*) associated with model marginal likelihood calculated using *fastSTRUCTURE* algorithm.

The simulation result from *fastSTRUCTURE* and the BIC score from *DAPC* suggested two genetic demes (*K* = 2, *BIC* = 581.08), based on 9315 nuclear SNPs (Figure 1 and Figure A2). The first genetic deme (East deme) includes populations mostly from the New England (MA and ME), East North Central (WI, IL, and IN), and West Central (KS, OK, and LA) regions. The second genetic deme (West deme) includes populations mostly from West North Central (MN, NE, and SD) and West Central (KS and OK) regions. The prairie cordgrass cultivar, ‘Red River’, was categorized into West deme, and New York cultivars ‘STP’ and ‘KST’ were placed in East deme. Populations from the two demes were largely separated by the border of mixed-prairie and tallgrass prairie as defined by Weaver [8] and mapped by Lauenroth et al. [66]. The populations collected in North Dakota, South Dakota, Nebraska, and Kansas are generally in the mixed prairie. The populations collected in Minnesota, Iowa, Missouri, and Illinois are mostly located in the tallgrass prairie.

**Figure 2:**
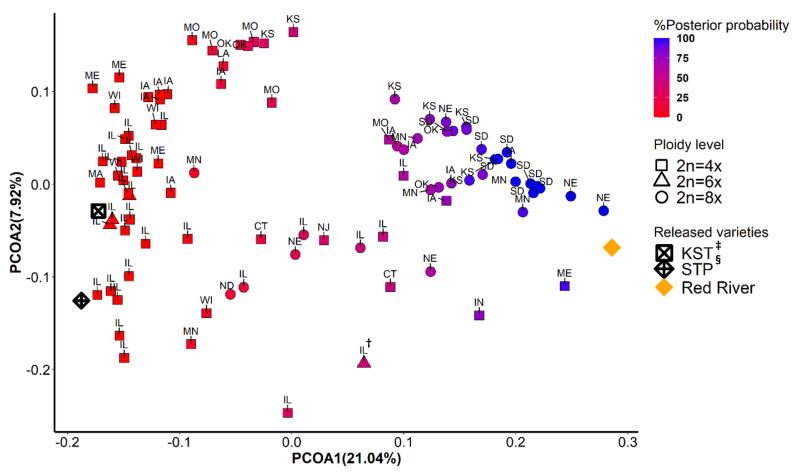
Principal coordinate analysis using 9315 SNPs data. The scores from the first (PCOA1) and the second (PCOA2) were plotted on *x*- and *y*-axis, respectively. The populations were colored in a gradient scale based on the posterior of probability assigned to two genetic groups inferred from *fastStructure* and *DAPC*. Shapes correspond to three levels of ploidy. ^†^: A hexaploid population collected from Illinois; ^‡^: KST = Kingston germplasm, Big Flats plant material center, NY; ^§^: STP = Southampton germplasm, Big Flats plant material center, NY.

**Figure 3:**
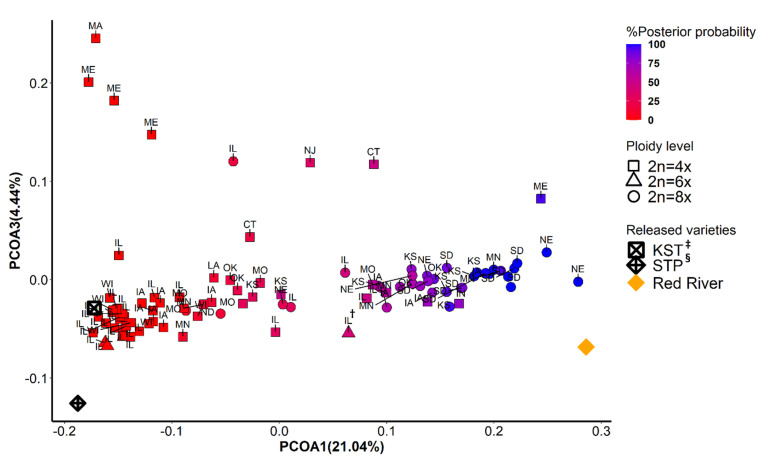
Principal coordinate analysis using 9315 SNPs data. The scores from the first (PCOA1) and the second (PCOA3) were plotted on *x*- and *y*-axis, respectively. The populations were colored in a gradient scale based on the posterior of probability assigned to two genetic groups inferred from *fastStructure* and *DAPC*. Shapes correspond to three levels of ploidy. ^†^: A hexaploid population collected from Illinois; ^‡^: KST = Kingston germplasm, Big Flats plant material center, NY; ^§^: STP = Southampton germplasm, Big Flats plant material center, NY.

**Figure A3:**
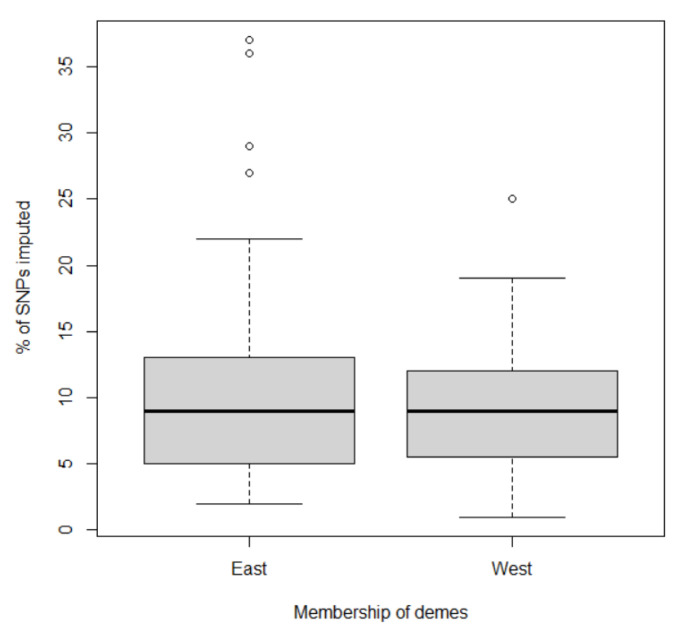
Box plot showing percentage of imputed SNPs for samples from two inferred demes using *fastSTRUCTURE* algorithm.

The first two principal coordinates separated East deme from West deme, indicating two major gene pools (Figure 2 and Figure 3). Although ploidy level is not fixed within these two gene pools, tetraploids (4x) and octoploids (8x) are primary cytotypes in East deme and West deme, respectively. In East deme, populations were more scattered on directions of both PCOA1 and PCOA2 compared to those in West deme (Figure 2). For example, populations collected from IA, OK, MO, and KS tended to form a subgroup separate from other populations. One octoploid populations from MN was clustered with the large IL group. Three octoploid populations from ND and NE located in the area between East deme and West deme on PCOA1. Three of the four hexaploid populations from IL clustered closely together and with most other IL populations, but one hexaploid population (marked as IL^†^) was considerably different. In West deme, populations were less variable/more tightly clustered than populations from East deme on PCOA1 and PCOA2. There were only four tetraploids in West deme. There were several populations from MO, NE, IA, IL, IN, CT, and KS that could equally likely be categorized in East deme or West deme. Furthermore, most of these populations are geographically adjacent to both populations from East deme and from West deme. These populations were also distributed along the mixed- to tallgrass prairie border. This provides support that populations from these states (MO, NE, IA, IL, and KS) are in areas where intraspecific breeding occurred. Populations from both demes were tightly distributed on PCOA3, except for eight populations from MA, ME, IL, NJ, and CT (Figure 3). Those populations could potentially be a subgroup from New England area. Differences in SNP missing rate could affect the results of population structure analysis. In our study, there was no significant difference between samples from two inferred demes for percentage of SNPs imputed (Kruskal–Wallis rank sum test: *p* = 0.77) Figure A3.

### 3.4. Analysis of Molecular Variance and Heterozygosity

Using 9315 SNP markers, analysis of molecular variance showed that SNP marker variance was significant among ploidy levels, among populations within ploidy levels, and among samples within populations (Table 1). Variance of SNP markers that accounted for ploidy levels, populations within ploidy levels, and sample within populations were 2.8%, 32.9%, and 64.3%, respectively. SNP marker variance was also significant among demes, ploidy levels within demes, and populations within ploidy levels. Deme, ploidy levels within demes, and populations within ploidy levels accounted for 14.3%, 4.6%, 81.1% of the SNP marker variance, respectively.

Average heterozygosity was calculated across a whole population panel, within each deme, and between two demes (Table 2). Average observed heterozygosity (*Ho*), average expected heterozygosity (*He*), and overall genetic diversity (*Ht*) across all populations were 0.27, 0.22, and 0.24, respectively. *Ho*, *He*, and *Ht* within East deme were 0.21, 0.19, and 0.20, respectively. *Ho*, *He*, and *Ht* within East deme were 0.35, 0.26, and 0.27, respectively. Inbreeding coefficients (*Fis*) across all populations, within East deme, and within West deme were −0.212, −0.133, and −0.369, respectively. The fixation index (*Fst*) across all populations, within East deme, within West deme, and between two demes were 0.05, 0.045, 0.053, and 0.079, respectively. Since a population-based imputation method (LD-kNNi) was used, the heterozygosity calculated in our study could be underestimated.

### 3.5. Mantel Tests and Canonical Correlation Analyses

The Mantel tests showed significant correlations of the genetic distance with both the environmental (*r* = 0.25) and the geographical distance (*r* = 0.33) (*p* < 0.001). The partial Mantel test generated a significant correlation between genetic and environmental distance (*r* = 0.068, *p* = 0.025) after controlling for geographical distance. This indicated that it is necessary to dissect the relationship between specific environmental variable and genetic distance. Following the Mantel and partial Mantel tests, CCA showed significant coefficients from five pairs of canonical variables (i.e., Canonical axes) (*p* < 0.01, Table 3), with correlations (*r*) ranged from 0.71 to 0.92. The top three canonical axes explained 73.5% variance cumulatively. Therefore, we selected the top three canonical axes and presented their correlations with genetic (PCOAs) and environmental/geographical variables in Table 4. The first three PCOAs (i.e., PCOA1, PCOA2, and PCOA3) showed significant correlations with canonical axes (II) (*r* = 0.618), (II) (*r* = 0.578), and (I) (*r* = 0.947), respectively. This indicated that populations separated by PCOA3 in PCOA were largely contributed by canonical variables on canonical axis (I). Populations separated by PCOA1 and PCOA2 were largely contributed by canonical variable on axis (II).

In canonical axis (I), LONG, MPDM, MPSP, and MPWI were significantly correlated with canonical axis (I) (*r* = 0.805, 0.601, 0.549, and 0.617, respectively), which resulted in separating East deme, West deme, and the New England populations (i.e., MA, ME, IL, NJ, and CT). The ALT and SDAT were also contributing to the pattern in a relatively low magnitude (r = −0.421 and −0.417, respectively). In canonical axis (II), ALT, MTWM, MPDM, and EF were significantly correlated with canonical axis (II) (*r* = 0.483, 0.426, −0.463, and 0.496, respectively), which resulted in separating East deme, West deme, and a widely scattered pattern in East deme populations. The LONG also showed a moderately high correlation with canonical axis (II) (*r* = −0.419). In canonical axis (III), a complex of environmental and geographical variables was correlated with PCOAs, especially PCOA7 and PCOA8. The LAT, MAT, MTWM, MTCM, MPSP, MPSU, MPAU, and MPWI were the top contributors in canonical axis (III). Notably, several precipitation variables were associated with LONG in canonical axis (I) while temperature variable were associated with LAT on canonical axis (II).

## 4. Discussion

### 4.1. Intraspecific Genetic Diversity

Using nuclear molecular markers, significant genetic diversity and population structures were found within perennial grass species [67,68,69]. In this study, analysis of molecular variance showed significant variance among ploidy levels and demes but accounted for only a small portion (2.78% and 14.32%, respectively) of the total variance. Large variance among and within populations indicated a greater landscape diversity in the population level in prairie cordgrass. This is congruent to results from a genetic diversity study on big bluestem (Andropogon gerardii Vitman) accessions by Price et al. [69]. Fragmentation of habitats and rhizome-preferred reproductive nature of prairie cordgrass could explain the significant divergence among populations and individuals [1]. We used fixation statistics such as *He*, *Ho*, *Fis*, and *Fst* to evaluate the degree of divergence within and among demes. The populations within West deme exhibited higher heterozygosity than that in East deme, as indicated by both the fixation statistics and the principal component analysis between and within demes. However, this is likely due to a larger number of octoploidy populations in the West Deme compared to that in the East Deme. Although dismoic SNPs calls were used for samples of all three ploidy levels, we still observe a higher heterozygosity level in the octoploidy dominant West Deme than that in the tetraploidy dominant East Deme. Similar to switchgrass, the two identified potential regional gene pools have a dominant ploidy level, either tetraploid or octoploid [70]. Compared to the phylogeograhic study using chloroplast sequences by Kim et al. [22], we included more populations and revealed different patterns of adaptation patterns among those populations. In this study, two major population demes were divided largely by the border of mix-grass prairie and tallgrass prairie. However, in the Kim et al. [22] study, the first chloroplast haplotype group had a wide longitudinal distribution that ranged from central Nebraska to the east coast of Maine. Prairie cordgrass is an efficient wind-pollinated species, population formation through bi-paternal introgression could be potentially detected using nuclear DNA. As chloroplast DNA is mediated through a uni-parental inheritance pattern, the populations from the first chloroplast haplotype group may represent one large ancestral ecotype with a wide distribution that later subdivided into two ecotypes as indicated by the SNP data from this study. The third chloroplast haplotype group distantly apart from the first group may indicate a historical migration of populations from south to north whenever they are able to adapt to different hardiness zones. The geographical barrier and mismatching ploidy levels may prevent further admixture between tetraploids and octoploids separated by the mix- and tallgrass prairie border. In early studies on switchgrass, upland-lowland switchgrass differential is largely latitudinal, caused by a combination of temperature and photoperiod [32,71]. However, recent studies indicated that the differences in ploidy levels also played an important role in restricting gene flow between potential genetically distinct groups [70,72,73]. In this study of prairie cordgrass, the gradient appears to be largely longitudinal. The wide dispersal of several populations from IL, IA, MN, ME, and WI could be due to human activities such as railroad transportation in the Central US regions or migratory trafficking from the west to east coasts as many of the natural populations were collected along the railway tracks [22,74,75].

### 4.2. Genetic and Geographical/Environmental Associations

Phenological and morphological differentiation due to geographic isolation and climatic gradients was observed within several tallgrass species native to North America [1,76,77,78,79]. In this study, we also detected significant influence of environmental condition on distribution of natural prairie cordgrass populations collected from the east and midwest US regions. The Mantel tests and CCA indicated a strong correlation of genetic distance and environmental/geographical variables among the prairie cordgrass populations. Our results also indicated that both LONG and ALT played important roles in forming a general separation of gene pools from east to west in prairie cordgrass. Geographical barriers such as the tall- and mixture prairie border in the USA, could create separate gene pools [80,81]. Precipitation patterns or moisture gradient also has an impact on the distribution of grass species [81,82,83]. In our study, precipitation variable such as MPDM, MPSP, and MPWI, together with LONG and ALT, were highly correlated with the genetic distance in the same canonical axis. This is not surprising since the origins of populations collected in this study are generally covered by the east-west decreasing precipitation gradients, which plays an important role in shaping the great plain grass prairie [84]. Temperature patterns in mid- and eastern US are largely governed by LAT and ALT [85]. This explains the high correlations of LAT with the temperature variables such as MAT, MTWM, MTCM, MTSP, MTSU, MTAU, and MTWI. However, LAT and temperature variables are mostly significant on canonical axis (III), which indicated that temperature has less impact on the genetic distance among prairie cordgrass populations compared to precipitation and ALT. As ALT correlates both precipitation and temperature in eastern US, it is expected that ALT was significantly correlated with climatic variables such as MAP, SDAT, MPDM, MPSP, and MTWM, MPDM, MTAU, MPWI on canonical axes (I) and (II), respectively. Ecoregion factor is highly correlated with canonical axis (II) which indicated that factors such as landforms, soils, vegetation, land use, wildlife, and hydrology also play important roles in shaping the prairie cordgrass distribution. However, ecoregion factor is also highly correlated with environmental factors. According to Bailey [86], the ecoregions were first defined by the largest units and successively subdivides them. At the continental level, temperature and precipitation are major factors in defining the large sections of ecoregions. In our study, we also found that the ecoregion factor showed the similar level of correlation with genetic variation as those for temperature and precipitation variables. In conclusion, geographical factors of LONG and ALT, and environmental factors of MAT, MAP, MTWM, MPDM, MPSP, and MPWI are most important in distinguishing the intraspecific distribution of prairie cordgrass.

## 5. Conclusions

Our research reported the population genomic variation and potential diversity centers in prairie cordgrass based on the analysis of 9315 SNPs from 96 natural populations. Two distinct genetic groups were identified which were associated with ploidy levels and geographical and ecotypic separation. Analysis of intraspecific variation among and within genetic groups and ploidy levels revealed evidence of adaptation history of prairie cordgrass in the Midwest and Eastern USA. The major gene flow in prairie cordgrass could be a consequence of geographic, climatic events, and human activity. Future studies on local landscape variation in prairie cordgrass could provide further information on the adaptation strategies in perennial grass species. From a standpoint of improving prairie cordgrass, both for biofuel production and conservation purpose, the identification of divergent genetic resource could provide opportunities to combine breeding value from different gene pools.

## Figures and Tables

**Table 1:** Analysis of molecular variance (AMOVA) for 96 prairie cordgrass populations based on hierarchical models. The first model consisted of ploidy levels, population within ploidy level, samples within population was calculated using 9315 SNPs data. The second model consisted of demes, ploidy levels within deme, and populations within ploidy level.

		DF ^†^	Sums of Squares	Mean Squares	Percentage of Variance Component
Model 1	Ploidy levels	2	4325	2162 **^,‡^	2.8
	Populations/ploidy level	93	101,614	1093 **	32.9
	Samples/populations/ploidy level	91	49,761	547 ***	64.3
Model 2	Demes	1	16,886	16,886 *	14.3
	Ploidy levels/demes	3	7073	2358 **	4.6
	Populations/Ploidy levels/demes	91	143,274	1574 **	81.1

^†^: Degrees of freedom varied across variables; ^‡^: * Significant at the *p* < 0.05, ** Significant at the *p* < 0.01, *** Significant at the *p* < 0.001.

**Table 2:** Genetic diversity of prairie cordgrass populations. Two demes were categorized-based fastStructure and DAPC of 9315 SNPs data. Heterozygosities were calculated using ‘hierfstat’ R package [51]; Fixation index was calculated using ‘hierfstat’ R package according to Weir & Cockerham [52].

	*N*	*Ho*	*He*	*Ht*	*Fis*	*Fst*
Overall	96	0.27	0.22	0.24	−0.212	0.050
East deme	61	0.21	0.19	0.20	−0.133	0.045
West deme	35	0.35	0.26	0.27	−0.369	0.053
Between two demes						0.079

*N*, number of individuals; *Ho*, observed heterozygosity; *He*, expected heterozygosity; *Ht*, overall gene diversity calculated from expected, observed heterozygosity, and number of individuals; *Fis*, inbreeding coefficient calculated from expected and observed heterozygosity; *Fst*, fixation index in overall, within each deme, and between two demes.

**Table 3:** Canonical correlation analysis of the PCOA and environmental/geographical variables in prairie cordgrass natural populations.

Canonical Axes	Canonical Correlation (*r*)	Variance Explained (*%*)	*F* Value	*p* Value (Prob > *F*)
I	0.92	37.8	3.5	<0.001
II	0.87	21	2.8	<0.001
III	0.83	14.7	2.3	<0.001
IV	0.78	10.9	1.9	<0.001
V	0.71	6.9	1.4	<0.01
VI	0.57	3.4	1.1	<0.33

*Canonical axes*, consisted of paired canonical variables; Canonical Correlation (*r*), correlations of POCA and environmental/geographical variable with canonical variables; Variance explained (%), percentage of variance explained by each pair of variables; *F* value, statistical test of canonical correlation coefficients (*F*-approximations of Wilks’ Lambda); *p* value (Prob > F), probability of the *F* values for statistical significance of canonical correlation coefficients.

**Table 4:** Canonical correlation analysis of the PCOA and environmental/geographical variables in prairie cordgrass natural populations.

	(I)	(II)	(III)
**Genetic**			
PCOA1	−0.123	0.618	−0.032
PCOA2	−0.178	0.578	−0.297
PCOA3	0.947	0.147	−0.143
PCOA4	−0.07	0.304	0.123
PCOA5	−0.193	−0.141	−0.358
PCOA6	−0.057	−0.01	−0.171
PCOA7	0.057	−0.035	−0.521
PCOA8	0.08	0.287	0.548
PCOA9	−0.016	−0.031	−0.297
PCOA10	−0.044	−0.256	0.241
**Environmental/Geographical ^†^**			
LAT	0.166	−0.183	−0.601
LONG	0.805	−0.419	−0.057
ALT	−0.421	0.483	0.135
MAT	−0.058	0.192	0.604
MAP	0.394	−0.291	0.179
SDAT	−0.417	−0.017	−0.499
SDAP	0.252	0.231	0.135
MTWM	−0.26	0.426	0.545
MTCM	0.154	0.195	0.608
MPWM	−0.052	0.096	−0.094
MPDM	0.601	−0.463	0.233
MTSP	0.042	0.186	0.592
MTSU	−0.337	0.164	0.561
MTAU	−0.207	0.327	0.588
MTWI	0.127	0.112	0.591
MPSP	0.549	−0.323	0.222
MPSU	0.158	−0.063	0.197
MPAU	−0.06	−0.108	−0.085
MPWI	0.617	−0.384	0.198
EF	0.181	0.496	−0.178

^†^: LAT = latitude; LONG = longitude; ALT = altitude; MAT = mean annual temperature; MAP = mean annual precipitation; SDAT = standard deviation of annual temperature; SDAP = standard deviation of annual precipitation; MTWM = mean temperature of the warmest month; MTCM = mean temperature of the coldest month; MPWM = mean precipitation of the wettest month; MPDM = mean precipitation of the driest month; MTSP = mean temperature of Spring; MTSU = mean temperature of Summer; MTAU = mean temperature of Autumn; MTWI = mean temperature of Winter; MPSP = mean precipitation of Spring; MPSU = mean precipitation of Summer; MPAU = mean precipitation of Autumn; MPWI = mean precipitation of Winter; EF = Ecoregion Factor.

## Data Availability

Raw sequencing reads were submitted to NCBI BioProject: PRJNA594199 Geographical and environmental data were submitted to the DRYAD repository (https://datadryad.org/stash/share/vd_e9EKUGtcnMVPYie42Na2dNGtiuoRa006H4A8WyHY, (accessed on 10 July 2021).

## Appendix A

**Table A1:** Ploidy levels, USDA hardiness zones (PHZ), Level III ecological regions of North America, average annual minimum temperature (°C), longitude and latitude of origin, missing data imputed (%), and membership of deme of 96 prairie cordgrass (*Spartina pectinata* Link) populations.

POP ID	State	*n*	Ploidy Level (x = 10)	Ecoregion ^†^	USDA Hardiness Zone ^‡^	Average Annual Minimum Temperature (°C)	Latitude	Longitude	Imputed (%) **	West Deme (%) ***	East Deme (%) ***	Deme Membership
PC09-102	CT	2	4x	NCZ	HZ7a	−17.8 to −15.0	41°21${}^{\prime }$0.09${}^{\prime \prime }$ N	71°54${}^{\prime }$33.08${}^{\prime \prime }$ W	19	48	52	East
PC19-101	IA	2	4x	WCBP	HZ5a	−28.9 to −26.1	41°55${}^{\prime }$7.77${}^{\prime \prime }$ N	92°34${}^{\prime }$57.55${}^{\prime \prime }$ W	3	0	100	East
PC19-102	IA	2	4x	WCBP	HZ5a	−28.9 to −26.1	41°56${}^{\prime }$23.29${}^{\prime \prime }$ N	92°34${}^{\prime }$35.82${}^{\prime \prime }$ W	3	0	100	East
PC19-103	IA	2	4x	WCBP	HZ5a	−28.9 to −26.1	42°0${}^{\prime }$29.81${}^{\prime \prime }$ N	93°25${}^{\prime }$38.27${}^{\prime \prime }$ W	2	0	100	East
PC19-105	IA	3	4x	WCBP	HZ5a	−28.9 to −26.1	42°39${}^{\prime }$42.72${}^{\prime \prime }$ N	94°13${}^{\prime }$36.54${}^{\prime \prime }$ W	5	0	100	East
PC19-106 *	IA	2	8x	WCBP	HZ5a	−28.9 to −26.1	43°4${}^{\prime }$58.56${}^{\prime \prime }$ N	94°26${}^{\prime }$52.32${}^{\prime \prime }$ W	17	57	43	West
PC19-107	IA	2	8x	WCBP	HZ5a	−28.9 to −26.1	43°5${}^{\prime }$5.98${}^{\prime \prime }$ N	94°32${}^{\prime }$14.99${}^{\prime \prime }$ W	8	77	23	West
PC19-108	IA	2	8x	WCBP	HZ5a	−28.9 to −26.1	42°19${}^{\prime }$48.21${}^{\prime \prime }$ N	96°19${}^{\prime }$37${}^{\prime \prime }$ W	13	100	0	West
PC19-109	IA	2	4x	WCBP	HZ5a	−28.9 to −26.1	42°12${}^{\prime }$20.6${}^{\prime \prime }$ N	96°15${}^{\prime }$5.22${}^{\prime \prime }$ W	9	13	87	East
PC19-110	IA	2	4x	WCBP	HZ5a	−28.9 to −26.1	41°47${}^{\prime }$33.84${}^{\prime \prime }$ N	96°2${}^{\prime }$33.19${}^{\prime \prime }$ W	6	74	26	West
PC19-111	IA	4	4x	WCBP	HZ5a	−28.9 to −26.1	42°1${}^{\prime }$27${}^{\prime \prime }$ N	93°43${}^{\prime }$6${}^{\prime \prime }$ W	6	0	100	East
PC19-112	IA	2	8x	WCBP	HZ5a	−28.9 to −26.1	42°1${}^{\prime }$55.93${}^{\prime \prime }$ N	94°27${}^{\prime }$19.83${}^{\prime \prime }$ W	4	63	37	West
IL-100	IL	2	4x	CCBP	HZ6a	−23.3 to −20.6	39°40${}^{\prime }$23.7${}^{\prime \prime }$ N	89°9${}^{\prime }$19.68${}^{\prime \prime }$ W	9	0	100	East
IL-102	IL	2	4x	CCBP	HZ5b	−26.1 to −23.3	40°3${}^{\prime }$55${}^{\prime \prime }$ N	88°14${}^{\prime }$19${}^{\prime \prime }$ W	27	0	100	East
IL-104	IL	2	4x	CCBP	HZ5b	−26.1 to −23.3	40°10${}^{\prime }$45${}^{\prime \prime }$ N	88°44${}^{\prime }$31${}^{\prime \prime }$ W	11	0	100	East
IL-105	IL	2	4x	CCBP	HZ5b	−26.1 to −23.3	40°54${}^{\prime }$41${}^{\prime \prime }$ N	87°56${}^{\prime }$36${}^{\prime \prime }$ W	14	30	70	East
IL-106	IL	2	4x	CCBP	HZ5b	−26.1 to −23.3	40°39${}^{\prime }$24${}^{\prime \prime }$ N	88°1${}^{\prime }$12${}^{\prime \prime }$ W	22	0	100	East
IL-99	IL	2	4x	CCBP	HZ6a	−23.3 to −20.6	39°45${}^{\prime }$ N	88°42${}^{\prime }$3${}^{\prime \prime }$ W	9	0	100	East
PC17-102	IL	3	6x	CCBP	HZ5b	−26.1 to −23.3	40°0${}^{\prime }$38.74${}^{\prime \prime }$ N	88°1${}^{\prime }$14.88${}^{\prime \prime }$ W	3	0	100	East
PC17-103	IL	2	6x	CCBP	HZ5b	−26.1 to −23.3	40°0${}^{\prime }$38.85${}^{\prime \prime }$ N	88°1${}^{\prime }$14.44${}^{\prime \prime }$ W	12	41	59	East
PC17-104	IL	2	6x	CCBP	HZ5b	−26.1 to −23.3	40°0${}^{\prime }$38.97${}^{\prime \prime }$ N	88°1${}^{\prime }$14.14${}^{\prime \prime }$ W	9	0	100	East
PC17-105	IL	2	4x	CCBP	HZ5b	−26.1 to −23.3	40°6${}^{\prime }$48.09${}^{\prime \prime }$ N	88°8${}^{\prime }$55.1${}^{\prime \prime }$ W	13	48	52	East
PC17-106	IL	2	8x	CCBP	HZ5b	−26.1 to −23.3	40°12${}^{\prime }$58${}^{\prime \prime }$ N	88°6${}^{\prime }$18${}^{\prime \prime }$ W	6	44	56	East
PC17-107	IL	2	4x	CCBP	HZ5b	−26.1 to −23.3	40°13${}^{\prime }$28.89${}^{\prime \prime }$ N	88°5${}^{\prime }$44.07${}^{\prime \prime }$ W	8	0	100	East
PC17-108	IL	2	4x	CCBP	HZ5b	−26.1 to −23.3	40°17${}^{\prime }$50.2${}^{\prime \prime }$ N	88°0${}^{\prime }$6.81${}^{\prime \prime }$ W	18	0	100	East
PC17-109	IL	2	4x	CCBP	HZ5b	−26.1 to −23.3	40°3${}^{\prime }$16.85${}^{\prime \prime }$ N	88°12${}^{\prime }$16.12${}^{\prime \prime }$ W	13	0	100	East
PC17-111	IL	3	4x	CCBP	HZ5a	−28.9 to −26.1	41°49${}^{\prime }$50.99${}^{\prime \prime }$ N	89°26${}^{\prime }$4.28${}^{\prime \prime }$ W	13	60	40	West
PC17-114	IL	2	4x	CCBP	HZ5b	−26.1 to −23.3	40°0${}^{\prime }$17.16${}^{\prime \prime }$ N	88°0${}^{\prime }$36.08${}^{\prime \prime }$ W	5	0	100	East
PC17-115 *	IL	2	4x	CCBP	HZ5b	−26.1 to −23.3	41°29${}^{\prime }$5.16${}^{\prime \prime }$ N	90°19${}^{\prime }$21.66${}^{\prime \prime }$ W	2	0	100	East
PC17-116	IL	2	6x	CCBP	HZ5b	−26.1 to −23.3	40°0${}^{\prime }$38.68${}^{\prime \prime }$ N	88°1${}^{\prime }$13.51${}^{\prime \prime }$ W	10	0	100	East
PC17-117	IL	2	8x	CCBP	HZ5b	−26.1 to −23.3	39°57${}^{\prime }$7.84${}^{\prime \prime }$ N	88°0${}^{\prime }$22.96${}^{\prime \prime }$ W	12	31	69	East
PC17-118	IL	2	4x	IRVH	HZ6a	−23.3 to −20.6	38°57${}^{\prime }$26.79${}^{\prime \prime }$ N	88°29${}^{\prime }$51.04${}^{\prime \prime }$ W	3	0	100	East
PC17-119	IL	2	4x	CCBP	HZ6a	−23.3 to −20.6	39°38${}^{\prime }$14.77${}^{\prime \prime }$ N	88°18${}^{\prime }$55.89${}^{\prime \prime }$ W	9	0	100	East
PC17-120	IL	2	4x	IRVH	HZ6a	−23.3 to −20.6	39°27${}^{\prime }$36.18${}^{\prime \prime }$ N	91°2${}^{\prime }$13.92${}^{\prime \prime }$ W	11	2	98	East
PC17-124	IL	2	4x	IRVH	HZ5b	−26.1 to −23.3	40°52${}^{\prime }$19.48${}^{\prime \prime }$ N	90°36${}^{\prime }$46.59${}^{\prime \prime }$ W	6	0	100	East
PC17-126	IL	2	4x	CCBP	HZ5b	−26.1 to −23.3	40°28${}^{\prime }$22.61${}^{\prime \prime }$ N	87°44${}^{\prime }$44.54${}^{\prime \prime }$ W	6	0	100	East
PC17-128	IL	4	4x	CCBP	HZ5b	−26.1 to −23.3	40°12${}^{\prime }$47.45${}^{\prime \prime }$ N	88°11${}^{\prime }$59.33${}^{\prime \prime }$ W	3	0	100	East
PC17-129	IL	2	4x	CCBP	HZ5b	−26.1 to −23.3	40°4${}^{\prime }$40.17${}^{\prime \prime }$ N	88°14${}^{\prime }$50.64${}^{\prime \prime }$ W	37	0	100	East
PC17-130	IL	4	4x	CCBP	HZ5b	−26.1 to −23.3	40°6${}^{\prime }$46.58${}^{\prime \prime }$ N	88°1${}^{\prime }$28.77${}^{\prime \prime }$ W	11	0	100	East
PC17-136	IL	2	4x	CCBP	HZ5b	−26.1 to −23.3	40°1${}^{\prime }$3.71${}^{\prime \prime }$ N	88°1${}^{\prime }$31.42${}^{\prime \prime }$ W	6	0	100	East
PC17-144	IL	2	8x	IRVH	HZ6a	−23.3 to −20.6	39°12${}^{\prime }$28.08${}^{\prime \prime }$ N	88°29${}^{\prime }$32.58${}^{\prime \prime }$ W	13	17	83	East
PC17-146	IL	2	4x	CCBP	HZ6a	−23.3 to −20.6	39°29${}^{\prime }$30.45${}^{\prime \prime }$ N	89°7${}^{\prime }$8.33${}^{\prime \prime }$ W	4	0	100	East
PC20-109 *	IL	2	8x	FH	HZ6a	−23.3 to −20.6	39°5${}^{\prime }$41.43${}^{\prime \prime }$ N	96°36${}^{\prime }$14.75${}^{\prime \prime }$ W	7	100	0	West
PC18-101	IN	2	4x	ECBP	HZ5b	−26.1 to −23.3	40°14${}^{\prime }$54.29${}^{\prime \prime }$ N	87°3${}^{\prime }$33.53${}^{\prime \prime }$ W	12	77	23	West
PC20-101	KS	2	8x	FH	HZ6a	−23.3 to −20.6	39°4${}^{\prime }$16.1${}^{\prime \prime }$ N	96°32${}^{\prime }$18.89${}^{\prime \prime }$ W	7	67	33	West
PC20-102	KS	2	4x	FH	HZ6b	−20.6 to −17.8	37°19${}^{\prime }$38.15${}^{\prime \prime }$ N	97°0${}^{\prime }$24.84${}^{\prime \prime }$ W	2	47	53	East
PC20-103	KS	2	8x	FH	HZ6a	−23.3 to −20.6	39°3${}^{\prime }$38.25${}^{\prime \prime }$ N	96°22${}^{\prime }$53.91${}^{\prime \prime }$ W	1	96	4	West
PC20-104 *	KS	2	8x	FH	HZ6b	−20.6 to −17.8	37°44${}^{\prime }$33.7${}^{\prime \prime }$ N	96°50${}^{\prime }$38.12${}^{\prime \prime }$ W	1	100	0	West
PC20-110	KS	2	8x	CGP	HZ6a	−23.3 to −20.6	38°54${}^{\prime }$32.13${}^{\prime \prime }$ N	97°14${}^{\prime }$44.54${}^{\prime \prime }$ W	3	92	8	West
PC22-101	LA	4	4x	SWTP	HZ8a	−12.2 to −9.40	32°53${}^{\prime }$54${}^{\prime \prime }$ N	91°59${}^{\prime }$27${}^{\prime \prime }$ W	9	12	88	East
PC25-101	MA	2	4x	NCZ	HZ6b	−20.6 to −17.8	42°33${}^{\prime }$37.2${}^{\prime \prime }$ N	70°55${}^{\prime }$18.96${}^{\prime \prime }$ W	5	0	100	East
PC23-101	ME	2	4x	APH	HZ5b	−26.1 to −23.3	43°55${}^{\prime }$13.93${}^{\prime \prime }$ N	69°51${}^{\prime }$49.57${}^{\prime \prime }$ W	11	95	5	West
PC23-102	ME	2	4x	APH	HZ5b	−26.1 to −23.3	44°16${}^{\prime }$4.57${}^{\prime \prime }$ N	69°1${}^{\prime }$0.65${}^{\prime \prime }$ W	9	0	100	East
PC23-103	ME	2	4x	APH	HZ5b	−26.1 to −23.3	44°29${}^{\prime }$26.19${}^{\prime \prime }$ N	68°1${}^{\prime }$1.51${}^{\prime \prime }$ W	18	0	100	East
PC23-104	ME	2	4x	APH	HZ5b	−26.1 to −23.3	44°31${}^{\prime }$39.58${}^{\prime \prime }$ N	67°53${}^{\prime }$14.11${}^{\prime \prime }$ W	9	0	100	East
PC27-101	MN	2	4x	LAP	HZ3b	−37.2 to −34.4	47°35${}^{\prime }$25.52${}^{\prime \prime }$ N	95°47${}^{\prime }$16.76${}^{\prime \prime }$ W	8	9	91	East
PC27-102 *	MN	2	8x	LAP	HZ4a	−34.4 to −31.7	47°48${}^{\prime }$40.55${}^{\prime \prime }$ N	96°36${}^{\prime }$38.84${}^{\prime \prime }$ W	5	70	30	West
PC27-103	MN	2	8x	LAP	HZ3b	−37.2 to −34.4	48°30${}^{\prime }$51.67${}^{\prime \prime }$ N	96°53${}^{\prime }$13.16${}^{\prime \prime }$ W	29	8	92	East
PC27-104	MN	2	8x	LAP	HZ4a	−34.4 to −31.7	46°40${}^{\prime }$27.03${}^{\prime \prime }$ N	96°25${}^{\prime }$30.67${}^{\prime \prime }$ W	9	100	0	West
PC27-106	MN	2	8x	WCBP	HZ4b	−31.7 to −28.9	45°9${}^{\prime }$5.72${}^{\prime \prime }$ N	95°57${}^{\prime }$41.39${}^{\prime \prime }$ W	12	94	6	West
PC27-108	MN	2	8x	WCBP	HZ4b	−31.7 to −28.9	44°32${}^{\prime }$36.86${}^{\prime \prime }$ N	94°17${}^{\prime }$41.97${}^{\prime \prime }$ W	11	67	33	West
PC29-101	MO	2	4x	CIP	HZ5b	−26.1 to −23.3	39°46${}^{\prime }$37.08${}^{\prime \prime }$ N	93°24${}^{\prime }$16.02${}^{\prime \prime }$ W	10	60	40	West
PC29-102	MO	2	4x	CIP	HZ6a	−23.3 to −20.6	39°45${}^{\prime }$35.76${}^{\prime \prime }$ N	92°41${}^{\prime }$16.86${}^{\prime \prime }$ W	4	14	86	East
PC29-103	MO	4	4x	CIP	HZ5b	−26.1 to −23.3	39°42${}^{\prime }$53.7${}^{\prime \prime }$ N	92°7${}^{\prime }$51.66${}^{\prime \prime }$ W	6	3	97	East
PC29-104 *	MO	2	4x	CIP	HZ6a	−23.3 to −20.6	37°51${}^{\prime }$42.63${}^{\prime \prime }$ N	94°13${}^{\prime }$37.97${}^{\prime \prime }$ W	4	29	71	East
PC29-106	MO	2	4x	CIP	HZ6b	−20.6 to −17.8	37°51${}^{\prime }$12.95${}^{\prime \prime }$ N	94°18${}^{\prime }$55.53${}^{\prime \prime }$ W	21	26	74	East
PC38-101	ND	2	8x	NGP	HZ4a	−34.4 to −31.7	47°27${}^{\prime }$33${}^{\prime \prime }$ N	98°49${}^{\prime }$58${}^{\prime \prime }$ W	18	14	86	East
PC31-101	NE	4	8x	CGP	HZ5b	−26.1 to −23.3	40°46${}^{\prime }$13.63${}^{\prime \prime }$ N	97°4${}^{\prime }$56.22${}^{\prime \prime }$ W	3	85	15	West
PC31-102	NE	2	8x	CGP	HZ5b	−26.1 to −23.3	40°44${}^{\prime }$28${}^{\prime \prime }$ N	99°33${}^{\prime }$35${}^{\prime \prime }$ W	9	100	0	West
PC31-103	NE	2	8x	CGP	HZ5a	−28.9 to −26.1	40°53${}^{\prime }$5.91${}^{\prime \prime }$ N	100°3${}^{\prime }$41.99${}^{\prime \prime }$ W	7	100	0	West
PC31-104	NE	2	8x	CGP	HZ5a	−28.9 to −26.1	41°2${}^{\prime }$22.28${}^{\prime \prime }$ N	100°25${}^{\prime }$19.84${}^{\prime \prime }$ W	12	27	73	East
PC31-105	NE	4	8x	CGP	HZ5a	−28.9 to −26.1	41°5${}^{\prime }$2.18${}^{\prime \prime }$ N	100°32${}^{\prime }$16.07${}^{\prime \prime }$ W	9	61	39	West
PC34-101	NJ	2	4x	ACPB	HZ6b	−20.6 to −17.8	40°0${}^{\prime }$10.56${}^{\prime \prime }$ N	74°37${}^{\prime }$8.49${}^{\prime \prime }$ W	17	34	66	East
PC20-105	NY	2	4x	CIP	HZ6b	−20.6 to −17.8	37°43${}^{\prime }$55.63${}^{\prime \prime }$ N	94°42${}^{\prime }$29.07${}^{\prime \prime }$ W	3	30	70	East
PC20-107	NY	2	8x	FH	HZ6a	−23.3 to −20.6	39°0${}^{\prime }$9.24${}^{\prime \prime }$ N	96°31${}^{\prime }$30.42${}^{\prime \prime }$ W	4	78	22	West
PC40-101	OK	2	4x	CIP	HZ6b	−20.6 to −17.8	36°51${}^{\prime }$32.52${}^{\prime \prime }$ N	94°54${}^{\prime }$47.76${}^{\prime \prime }$ W	8	18	82	East
PC40-102 *	OK	2	4x	CIP	HZ6b	−20.6 to −17.8	36°52${}^{\prime }$25.5${}^{\prime \prime }$ N	95°0${}^{\prime }$45.24${}^{\prime \prime }$ W	4	23	77	East
PC40-103	OK	2	8x	CGP	HZ7a	−17.8 to −15.0	36°49${}^{\prime }$43.56${}^{\prime \prime }$ N	97°4${}^{\prime }$3.47${}^{\prime \prime }$ W	2	89	11	West
PC40-104	OK	2	8x	CGP	HZ7a	−17.8 to −15.0	36°49${}^{\prime }$43.92${}^{\prime \prime }$ N	97°4${}^{\prime }$3.3${}^{\prime \prime }$ W	10	75	25	West
PC46-101	SD	2	8x	WCBP	HZ4b	−31.7 to −28.9	43°40${}^{\prime }$26.68${}^{\prime \prime }$ N	96°48${}^{\prime }$41${}^{\prime \prime }$ W	25	78	22	West
PC46-102	SD	2	8x	NGP	HZ4b	−31.7 to −28.9	43°32${}^{\prime }$5.52${}^{\prime \prime }$ N	96°49${}^{\prime }$50.69${}^{\prime \prime }$ W	7	100	0	West
PC46-103	SD	2	8x	NGP	HZ4b	−31.7 to −28.9	43°26${}^{\prime }$26.69${}^{\prime \prime }$ N	96°49${}^{\prime }$34.73${}^{\prime \prime }$ W	14	100	0	West
PC46-104 *	SD	2	8x	NGP	HZ4b	−31.7 to −28.9	43°23${}^{\prime }$17.41${}^{\prime \prime }$ N	96°49${}^{\prime }$34.67${}^{\prime \prime }$ W	4	95	5	West
PC46-105	SD	2	8x	NGP	HZ4b	−31.7 to −28.9	43°10${}^{\prime }$34.77${}^{\prime \prime }$ N	96°49${}^{\prime }$32.57${}^{\prime \prime }$ W	10	99	1	West
PC46-106	SD	2	8x	NGP	HZ4b	−31.7 to −28.9	42°58${}^{\prime }$1.2${}^{\prime \prime }$ N	96°49${}^{\prime }$34.73${}^{\prime \prime }$ W	19	86	14	West
PC46-107	SD	2	8x	NGP	HZ5a	−28.9 to −26.1	42°48${}^{\prime }$11${}^{\prime \prime }$ N	96°49${}^{\prime }$35.19${}^{\prime \prime }$ W	12	85	15	West
PC46-108	SD	2	8x	NWGP	HZ4b	−31.7 to −28.9	43°56${}^{\prime }$39.15${}^{\prime \prime }$ N	98°16${}^{\prime }$17.77${}^{\prime \prime }$ W	12	100	0	West
PC46-109	SD	2	8x	SCP	HZ5a	−28.9 to −26.1	43°26${}^{\prime }$55.46${}^{\prime \prime }$ N	100°1${}^{\prime }$41.2${}^{\prime \prime }$ W	11	100	0	West
PC55-101	WI	3	4x	WCBP	HZ5a	−28.9 to −26.1	43°31${}^{\prime }$27${}^{\prime \prime }$ N	89°29${}^{\prime }$51${}^{\prime \prime }$ W	11	0	100	East
PC55-102	WI	2	4x	NCHF	HZ4b	−31.7 to −28.9	44°3${}^{\prime }$12.62${}^{\prime \prime }$ N	90°5${}^{\prime }$23.37${}^{\prime \prime }$ W	12	10	90	East
PC55-103	WI	2	4x	NCHF	HZ4b	−31.7 to −28.9	44°39${}^{\prime }$40.94${}^{\prime \prime }$ N	91°3${}^{\prime }$14.96${}^{\prime \prime }$ W	36	0	100	East
PC55-104 *	WI	4	4x	NCHF	HZ4a	−34.4 to −31.7	45°30${}^{\prime }$21.77${}^{\prime \prime }$ N	92°1${}^{\prime }$12.12${}^{\prime \prime }$ W	5	0	100	East
PC55-105	WI	2	4x	DA	HZ4b	−31.7 to −28.9	43°26${}^{\prime }$46.02${}^{\prime \prime }$ N	90°46${}^{\prime }$48.11${}^{\prime \prime }$ W	18	0	100	East
KST ^¶^	-	-	4x	-	-	-	-	-	11	0	100	East
Red River	-	-	8x	-	-	-	-	-	12	100	0	West
STP *^§^	-	-	4x	-	-	-	-	-	9	0	100	East

^†^: APH = Acadian Plains and Hills, ACPB = Atlantic Coastal Pine Barrens, CCBP = Central Corn Belt Plains, CGP = Central Great Plains, CIP = Central Irregular Plains, DA = Driftless Area, ECBP = Eastern Corn Belt Plains, FH = Flint Hills, IRVH = Interior River Valleys and Hills, IRVH = Lake Agassiz Plain, NCHF = North Central Hardwood Forests, NCZ = Northeastern Coastal Zone, NGP = Northern Glaciated Plains, NWGP = Northwestern Great Plains, SCP = South Central Plains, SWTP = Southeastern Wisconsin Till Plains, WCBP = Western Corn Belt Plains [54] (available at www.epa.gov/wed/pages/ecoregions.htm, accessed on 17 July 2018). ^‡^: PRISM Climate Group – Oregon State University (2012). ^§^: STP = Southampton germplasm, Big Flats plant material center, NY. ^¶^: KST = Kingston germplasm, Big Flats plant material center, NY. *: Samples selected for creating pseudo-reference genome. **: Percentage of SNPs imputed using LD-kNNi algorithm. ***: Deme membership inferred using fastSTRUCTURE algorithm.
